# Study of the Effect of Distance and Misalignment between Magnetically Coupled Coils for Wireless Power Transfer in Intraocular Pressure Measurement

**DOI:** 10.1155/2014/692434

**Published:** 2014-07-06

**Authors:** Adrian E. Rendon-Nava, J. Alejandro Díaz-Méndez, Luis Nino-de-Rivera, Wilfrido Calleja-Arriaga, Felix Gil-Carrasco, Daniela Díaz-Alonso

**Affiliations:** ^1^Graduate Department, National Polytechnic Institute of Mexico (IPN), ESIME UPC, Avenida Santa Ana 1000, San Francisco Culhuacan, 04260 Mexico City, DF, Mexico; ^2^Electronics Department, INAOE, Luis Erro 1, 72840 Santa Maria Tonantzintla, PUE, Mexico; ^3^APEC, The Association to Avoid Blindness in Mexico, Hospital Luis Sanchez Bulnes, Vicente Garcia Torres 46, 04030 Coyoacan, DF, Mexico

## Abstract

An analysis of the effect of distance and alignment between two magnetically coupled coils for wireless power transfer in intraocular pressure measurement is presented. For measurement purposes, a system was fabricated consisting of an external device, which is a Maxwell-Wien bridge circuit variation, in charge of transferring energy to a biomedical implant and reading data from it. The biomedical implant is an RLC tank circuit, encapsulated by a polyimide coating. Power transfer was done by magnetic induction coupling method, by placing one of the inductors of the Maxwell-Wien bridge circuit and the inductor of the implant in close proximity. The Maxwell-Wien bridge circuit was biased with a 10 MHz sinusoidal signal. The analysis presented in this paper proves that wireless transmission of power for intraocular pressure measurement is feasible with the measurement system proposed. In order to have a proper inductive coupling link, special care must be taken when placing the two coils in proximity to avoid misalignment between them.

## 1. Introduction

Regarding power transfer in biomedical implants, two options exist to accomplish this: either the implant must have an internal battery to supply power or the energy must come from an outer source. The choice for power transfer method depends on the type of implant. Pacemakers [1, 2] and cochlear implants [3, 4], for example, can have an internal battery running the electronic circuitry without a problem. In the case of intraocular pressure (IOP) measurement implants, however, the use of a battery to power the system is not recommended because of the bulky nature of the battery and because the human eye is such a delicate organ. Nowadays, intraocular pressure measurement is mostly performed in the facilities of the nearest hospital with the aid of specialized medical equipment such as tonometers. In a conventional tonometer, intraocular pressure is inferred from the force required to flatten a constant area of the cornea. Other methods such as multifocal electroretinography have been investigated in order to develop a new mfERG-paradigm glaucoma analysis protocol and to gain a better understanding of glaucoma [5], however, there is not still an IOP micro system implanted inside the ocular glove to measure continuously IOP to understand glaucoma etiology. All methods developed for intraocular pressure measurement have a common disadvantage: the patient must travel to the hospital facility in order to get his pressure measured. This is the main reason why there has been continuously increasing research in recent years to develop a wireless intraocular pressure sensor system.

In the last decade, there has been a lot of research in order to develop a fully implantable system to monitor intraocular pressure. Most of these efforts propose an intraocular pressure implant to be placed over the sclera [6]. These approaches, however, may suffer from having intraocular pressure measurements with errors because the hardness of the sclera is different on every patient so the system could measure a normal intraocular pressure whereas in reality the patient could have an elevated intraocular pressure value. In [7], the authors propose to place the implant inside the eye (at the pars plana or on the iris). This approach solves the problem of measuring over the sclera; nevertheless, it poses another challenge with respect to magnetic coupling since now there will be more organic tissue between the coils.

Several wireless biomedical implants have been proposed in the literature. Most of them use magnetic induction coupling to power a CMOS circuit embedded in the implant [8–12]. The location of the implant inside the body of the patient and that of the external reader is of critical importance to achieve the maximum power in the coil of the implant. Both coils need to be placed as close to each other as possible; also they need to be parallel to each other and their centers need to be collinear.

This paper analyzes the effect of distance and alignment between two magnetically coupled coils for wireless power transfer in intraocular pressure measurement, characterizing the variations in the impedance of the reader coil by changing the distance between coils in a range from 2.0 cm to 0.1 cm and by misaligning them. In both measurements the maximum distance at which the coils could be separated without compromising the inductive coupling link was obtained. The measurements were performed using two different transmission mediums: air and a combination of air and porcine organic tissue. Two different types of transmission mediums were used in the experiments with porcine organic tissue. One transmission medium consisted of 1 mm of pork skin and 1 mm of fat. For the second transmission medium, 1 mm of pork skin, 1 mm of fat, and 2 mm of muscle were used.

For the implant, we propose a passive RLC tank circuit encapsulated by a polyimide coating. Using a passive circuit as the implant will significantly reduce power consumption demand since no CMOS circuitry will be required [13–15]. For the reader circuit, a variation of the Maxwell-Wien bridge circuit was proposed. The Maxwell-Wien bridge circuit was biased with a 10 MHz sinusoidal signal, which complies with the Mexican Federal Communications Commission regulation for free public radioelectrical radiation.

## 2. Methods

### 2.1. Magnetic Induction Coupling Method

**Figure 1:**
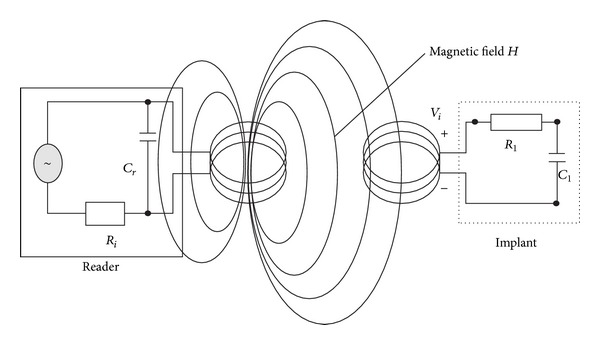
Transmission in a magnetic induction coupling system.

When a time-varying current circulates on the coil from the reader device, an electromagnetic field is created which in turn penetrates the cross-sectional area of the coil of the implant (Figure 1).

The wavelength of the frequency range used for the reader coil is generally several times greater than the distance between the coils so the electromagnetic field may be treated as a simple magnetic alternating field. A small part of the emitted field penetrates the coil of the implant, which is some distance away from the coil of the reader device. A voltage *V*_*i*_ is generated in the coil of the implant by inductance. A capacitor *C*_*r*_ is connected in parallel with the coil of the reader; the capacitance of *C*_*r*_ is selected in such a way that it can work with the inductance of the coil from the reader to form a parallel resonant circuit with a resonant frequency corresponding with the transmission frequency of the reader [16]. The system shown in Figure 1 basically works as Close Coupling RFID Systems work; the reader device provides the transponder (the implant in our case) with energy and the transponder transmits data to the reader.

### 2.2. Sensor Structure

**Figure 2:**
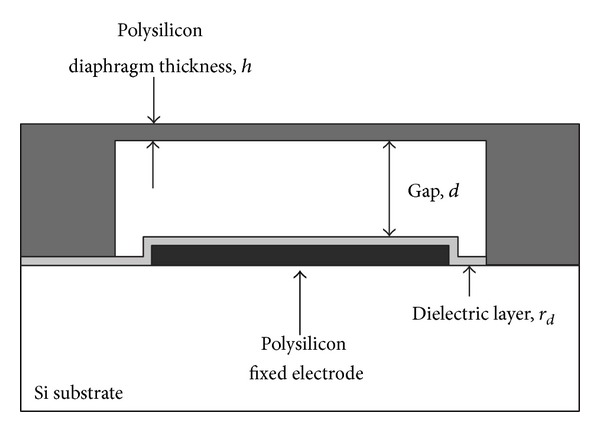
Structure of MEMS touch mode capacitive pressure sensor.

The IOP sensor will be a MEMS touch mode capacitive pressure sensor (MEMS TMCPS). The basic element of a TMCPS is an equivalent parallel plate capacitor with clamped edges [17] as shown in Figure 2. The capacitance *C* is represented as

(1) $$\begin{array}{c} C=\frac{\varepsilon A}{d}, \end{array}$$

where *ε* is the permittivity of the dielectric thin film, *A* is a touching area of diaphragm, and *d* is the gap space between the two electrodes. The upper plate of the capacitor, known as the* diaphragm*, will deform in response to an applied pressure and finally will come into contact with the dielectric thin film. Since the permittivity and the thickness are constant values, the capacitance *C* will only depend on the touching area *A*. Consequently we can measure the applied pressure to the diaphragm by reading the capacitance.

Let us consider a circular capacitive membrane. If the pressure acting on a circular plate is uniformly distributed all over the plate, all equidistant points from the center of the plate will have the same deflections. Solving the differential equation for a circular plate clamped on its periphery [18], we have that the deflection experienced by the plate varies with pressure according to

(2) $$\begin{array}{c} w\left(r\right)=\left(\frac{3P\left(1-v^{2}\right)}{16Eh^{3}}\right)\left[a^{2}-r^{2}\right], \end{array}$$

where *w*(*r*) is a function of the radius *r* of the membrane, *P* is the applied pressure in Pascals, *v* is the rate of Poisson for the material (in this case Polysilicon), *E* is the Modulus of Young for the material, *h* is the thickness of the plate, and *a* is the radius of the plate.

Once we know how much the membrane plate will be deflected with pressure variations, it is still necessary to calculate how pressure variations will reflect in changes in the value of capacitance of the MEMS IOP sensor. Applying a surface integral all over the capacitor plate [19], the capacitance will behave as

(3) $$\begin{array}{c} C=2\pi \varepsilon \overset{a}{\underset{0}{\int }}\frac{rdr}{\left(d-w\left(r\right)\right)}, \end{array}$$

where, as mentioned earlier, *w*(*r*) is the function of deflection depending on the radius *r* of the membrane, *a* is the radius of the plate, *d* is the distance between plates when no pressure is applied, and *ε* is the relative permittivity of the material with respect to vacuum.

**Figure 3:**
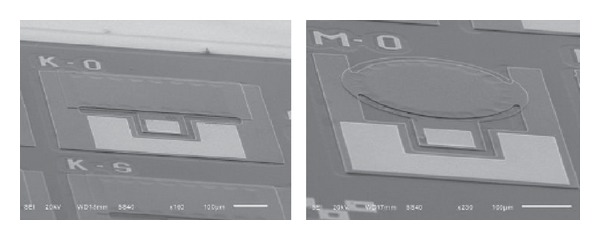
MEMS capacitive sensor prototypes, fabricated at the National Institute of Astrophysics, Optics and Electronics.

Several MEMS capacitors prototypes have already been fabricated at the facilities of the National Institute of Astrophysics, Optics and Electronics in Puebla, Mexico, as shown in Figure 3 [20]. A Touch mode capacitive pressure structures were fabricated by using a three-layer Polysilicon surface micromachining process called PolyMEMS-INAOE [21]. The main advantage of the fabrication process is its capability of building a suspended diaphragm over a cavity by removing sacrificial material due to a novel design. The design of MEMS IOP sensor includes a set of holes along two opposite diaphragm sides to allow the flow of the etch solution, whereas the other sides are fully clamped. At the same time it is possible to keep the cavity closed, with a subsequent sealing step, without the necessity of bonding techniques to form the final device. The variation range in capacitance of the sensor is of approximately 2 pF. Considering the dimensions of the sensor (600 *μ*m in diameter/side) such as active area, distance between plates, and the thickness of the bending plate, the variation range in capacitance of the MEMS sensor corresponds to a variation in pressure of 10 mm Hg to 80 mm Hg.

### 2.3. Equivalent Impedance of the Coil

**Figure 4:**
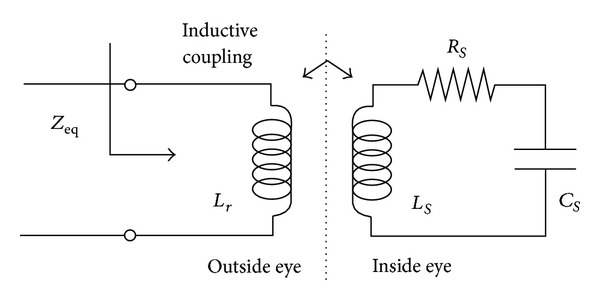
Equivalent circuit of an electrical model for an RLC circuit.

Figure 4 shows an electrical model for an RLC tank circuit coupled with an external coil. The coil *L*_*s*_ of the implant and the capacitor *C*_*s*_ form a resonant circuit tuned to the transmission frequency of the reader.

The implant resonating frequency and its coupling to the reader coil can be modeled with a two-port network. The input impedance of the reader coil is expressed with electrical parameters of the implant it encloses. By using two-port network analysis, laws of Kirchhoff, and taking into account that *s* = *jw* = *j*2*πf*, from Figure 4 we have

(4) $$\begin{array}{c} V_{r}=sL_{r}I_{r}+sL_{m}I_{s},\\ V_{s}=sL_{m}I_{r}+sL_{s}I_{s}, \end{array}$$

where *V*_*r*_ and *I*_*r*_ are the voltage and current on the reader coil, *V*_*s*_ and *I*_*s*_ are the voltage and current on the implant coil, and *L*_*r*_, *L*_*s*_, and *L*_*m*_ are reader inductance, sensor inductance, and mutual inductance, respectively. By circuit analysis, the equivalent impedance *Z*_eq_ in Figure 4 [22] seen from the reader is

(5) $$\begin{array}{c} Z_{\text{eq}}=\frac{V_{r}}{I_{r}}=j2\pi fL_{r}\left[1+k^{2}\frac{{\left(f/f_{s}\right)}^{2}}{1-{\left(f/f_{s}\right)}^{2}+\left(1/Q\right)j\left(f/f_{s}\right)}\right], \end{array}$$

where *f* is the excitation frequency, *k* is the coupling factor (totally dependent on physical geometries such as the area of the implant and the reader coils and the separation distance between them), *f*_*s*_ is the resonating frequency of the implant, defined by

(6) $$\begin{array}{c} f_{s}=\frac{1}{2\pi }\left(\frac{1}{\sqrt{L_{s}C_{s}}}\right) \end{array}$$

and *Q* is the quality factor of the sensor at resonance, given by

(7) $$\begin{array}{c} Q=\frac{2\pi f_{s}L_{s}}{R_{s}}. \end{array}$$

It can be seen from (5) that in order to change the coil impedance *Z*_eq_ from the reader, one must change either the coupling factor *k* or the resonating frequency *f*_*s*_ of the implant.

For intraocular pressure applications, the distance between coils will remain constant so the coupling factor *k* will not change, making the resonating frequency *f*_*s*_ of the implant the only variable capable of changing the impedance of (5).

The changes in capacitance of the IOP sensor due to changes in the applied pressure will change the RLC circuit resonating frequency. Such changes in the resonating frequency of the implant will be detected in the reader coil as variations in the coil impedance.

### 2.4. Maxwell-Wien Bridge Circuit

**Figure 5:**
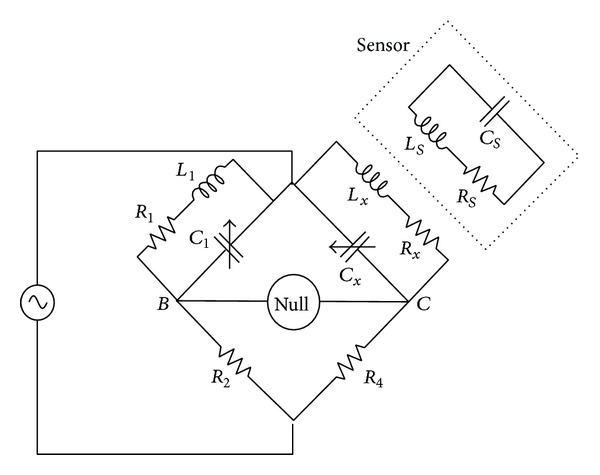
Maxwell-Wien circuit variation proposed for energy and data transfer.

The circuit proposed to receive data and to transfer power to the implant is a Maxwell-Wien bridge circuit variation [23], shown in Figure 5. Resistors *R*_2_ and *R*_4_ are of the same value to bring balance to the circuit. In the absence of the implant, that is, when the two coils *L*_*s*_ and *L*_*x*_ are far enough from each other, the Maxwell-Wien bridge will be balanced; in other words, the voltage between nodes *B* and *C* will be zero.

When the coil *L*_*s*_ of the implant begins to approach the reader coil *L*_*x*_, the coupling factor *k* value will be higher, therefore changing the impedance value of the coil *L*_*x*_. This will in turn make the voltage between nodes *B* and *C* nonzero.

If, on the other hand, the coil of the implant *L*_*s*_ is fixed at a certain distance near *L*_*x*_, the value of the coupling factor *k* will remain constant. The variations in the resonating frequency of the RLC circuit of the implant will reflect in changes in the impedance of the reader coil *L*_*x*_. Such variations in the impedance of *L*_*x*_ will change the voltage between nodes *B* and *C* of the Maxwell-Wien bridge circuit.

### 2.5. Implant Location and Connection

To determine the location of the implant within the eye is not an easy task since it depends on both medical and technological factors. Among the medical factors that come into play to determine the most convenient location of the implant are the following.

- The location of the implant must not obstruct vision.
- Surgery must not be too invasive.
- The location of the implant must not limit the movement of the eye.

With respect to technological restrictions for implant location we can find the following.

- Location of the implant shall have the greatest possible area in order to maximize magnetic coupling between coils.
- The location of the implant must be as near as possible from the exterior of the human body so that, as mentioned previously, the maximum magnetic coupling can be obtained.

**Figure 6:**
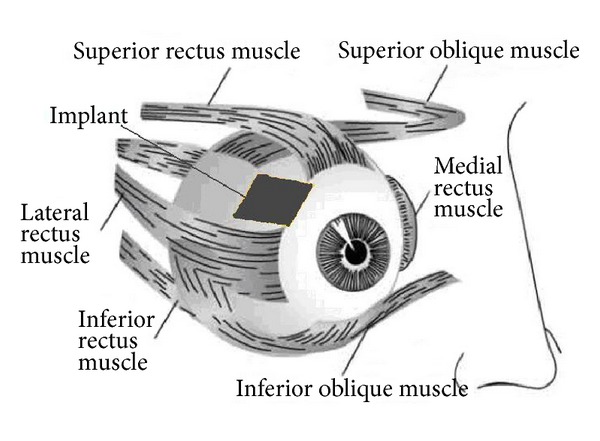
Proposed location for the implant (taken from [24]).

As a result of the discussions held with the CEO of Luis Sanchez Bulnes Hospital in Mexico City, Dr. Felix Gil Carrasco, it was proposed that the implant should be located in the section of the eye shown in Figure 6, between the superior rectus and lateral rectus muscles.

One of the main reasons why the coil and the MEMS membrane will not be fabricated in the same fabrication process is due to the rigidity required by the capacitor. As we know, the coil will be fabricated over a flexible substrate (polyimide) which will allow it to conform to the curvature of the human eye. The MEMS capacitor, on the other hand, requires that the capacitive plate which will not be in contact with the intraocular pressure remains rigid, as a reference for the bending plate which will be deformed with applied pressure. By the aforementioned it was decided to fabricate the MEMS pressure sensor over a silicon substrate.

**Figure 7:**
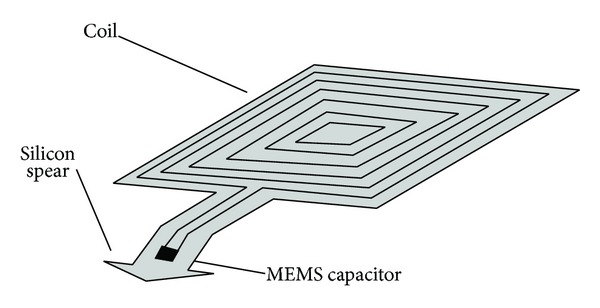
Implant geometry and interconnection.

Another reason to separate fabrication processes, not less important than the previous one, is because of practical purposes when surgically inserting the implant. The ultimate goal of this study is to develop an IOP sensor system capable of measuring pressure directly into the aqueous humor to avoid possible measurement errors caused by the hardness of the sclera which varies from patient to patient. For the implant to be able to measure IOP directly into the aqueous humor, the MEMS sensor will be located in a small appendix next to the coil which will be inserted into the eyeball so it can be in direct contact with the aqueous humor. The implant coil, however, will remain over the sclera, allowing it to have a better magnetic coupling with the reader coil.  Figure 7 shows the geometry of the implant.

The coil will be attached to the sclera by sutures at the corners of the implant or by some organic glue to avoid movement. In the case of the sensor it will also be necessary to guarantee that it will remain fixed to prevent it from coming out of the eyeball. The silicon substrate of the sensor will play a double role since besides serving as a mechanical support for one of the capacitor plates, it will also serve to avoid that the sensor comes out of its position once inserted in the eye. The substrate will be cut in a spear-like geometry to achieve the latter. The substrate (MEMS sensor included) will be expected to have dimensions of 2 mm × 2 mm.

Once the MEMS sensor is attached to the polyimide substrate, the coil and the sensor will be connected by Aluminum deposition. The last step of the process will be to encapsulate the implant with a polyimide coating.

## 3. Results and Discussion

### 3.1. External Device and Implant Fabrication

A variation from the Maxwell-Wien bridge circuit was fabricated on an FR4 PCB. To avoid degradation of the input and output signals, SMA connectors and cables from the circuit to both the measurement and powering equipment were used. The Maxwell-Wien bridge was powered with an AWG2041 Arbitrary Waveform Generator. The output was measured with a Tektronix TDS3054 Oscilloscope.

The input signal frequency was chosen to be in the 10 MHz range in order to comply with the Mexican Federal Communications Commission regulation for free public radioelectrical radiation. Another important reason for the chosen frequency range is because of the results published in a recent research which show that for a short time radiation emission in the 10 MHz frequency range, other medical implants such as pacemakers, implantable cardioverter defibrillators, and implantable neurostimulator devices remain unaffected [25].

High-frequency surface mount resistors and capacitors were used to build the Maxwell-Wien circuit. The inductors (*L*_1_ and *L*_*x*_ from Figure 4) were fabricated along with the PCB as planar coils.  Table 1 shows the values of the components from the Maxwell-Wien bridge. The diameter of the coils was designed to be greater than the diameter of the implant coil on purpose. The latter was done to reduce the risk of decoupling between coils due to misalignment.

**Figure 8:**
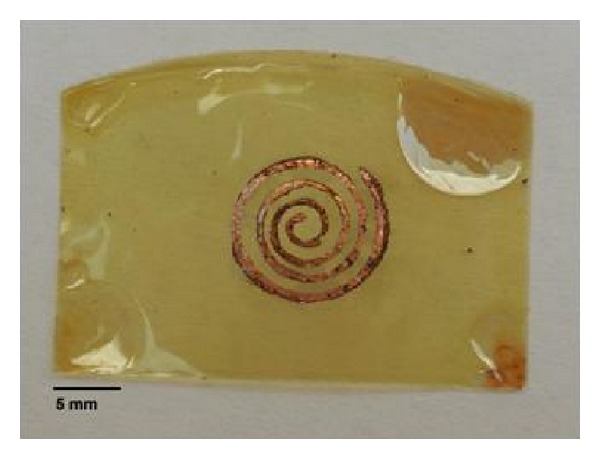
Copper coil with polyimide coating.

The implant was fabricated in the Microelectronics Laboratory facility from the National Institute of Astrophysics, Optics and Electronics (INAOE). A 7.5 *μ*m layer of HD Microsystems PI-2610 polyimide was deposited on a silicon wafer and then cured as advised by the manufacturer. A 30 *μ*m thick copper coil was then placed over the polyimide layer and a final 7.5 *μ*m layer of polyimide was deposited on top to fully encapsulate the inductor. The device was taken into a final curing process and then released from the silicon wafer as shown in Figure 8.

**Figure 9:**
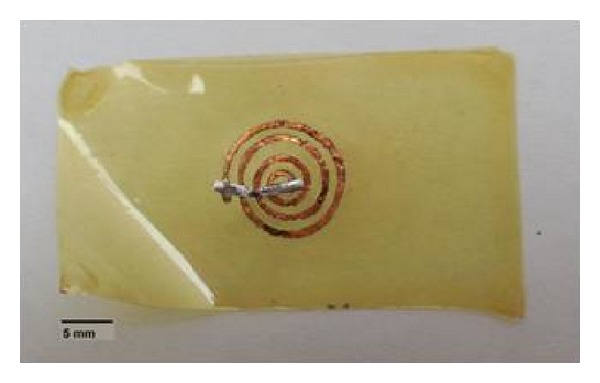
Passive RLC tank circuit with polyimide coating.

To improve the quality factor of the implant coil and its response at the 10 MHz range, polyimide was removed from the ends of the coil to solder a surface mount capacitor of 2.7 nF, as shown in Figure 9. The implant coil was designed to have an inner radius of 1.2 mm, conductor width of 0.5 mm, spacing between turns of 0.9 mm, and a number of turns equal to 4. The total diameter of the coil used was 12 mm.

### 3.2. Measurements

**Figure 10:**
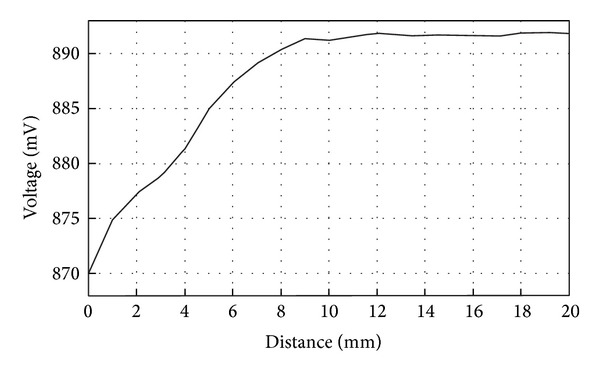
Voltage variations on reader coil versus distance. Transmission medium between coils: air.

The implant coil was placed parallel to one of the coils of the Maxwell-Wien circuit to measure the mutual magnetic induction coupling between them. A sweep was made by changing the distance between coils, starting at 20 mm and ending at 1 mm. The transmission medium between coils for these measurements was solely air.  Figure 10 shows voltage variations on the reader coil due to changes in mutual coupling at a frequency of 10 MHz.

**Figure 11:**
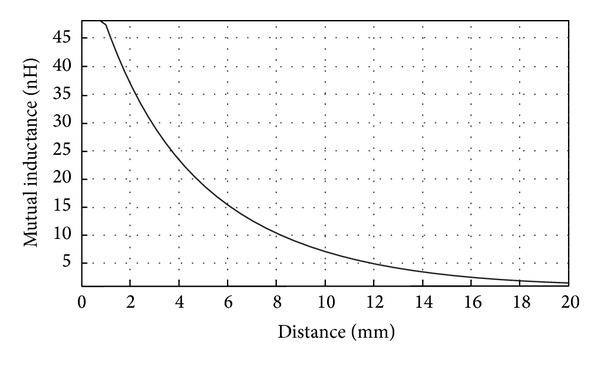
Mutual inductance versus distance. Transmission medium between coils: air.

As it can be seen from Figure 10, the variations in voltage started to occur when the distance between coils was below approximately 8 mm. It is clear that for the system we proposed there was practically no magnetic induction when the distance between coils was beyond 10 mm since there were no voltage variations. For intraocular measurement, the distance between coils is a fixed variable, so in order to obtain a reliable measurement it is, therefore, of great importance to make sure that the distance between them will remain constant between measurements to avoid possible errors on the readings.  Figure 11 shows the calculated mutual inductance between coils versus the distance between them.

**Figure 12:**
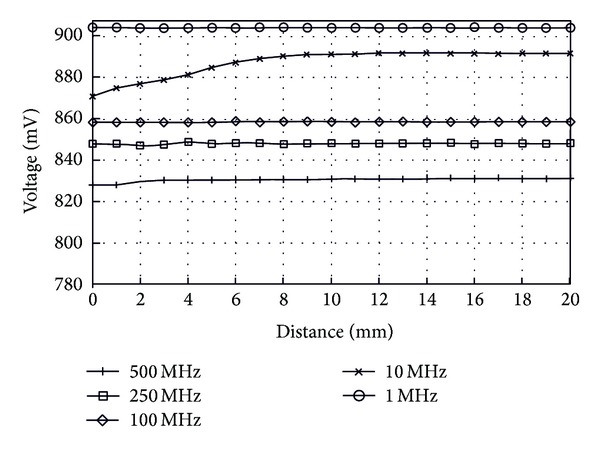
Voltage variations on reader coil versus distance. Transmission medium between coils: air. Measurements were done for various frequencies (1 MHz, 10 MHz, 100 MHz, 250 MHz, and 500 MHz).

Besides the chosen frequency of 10 MHz and maintaining air as the transmission medium, coupling between the implant and the external reader at various frequencies was measured as seen in Figure 12. As before, a sweep was made by changing the distance between coils, starting at 20 mm and ending at 1 mm for 500 MHz, 250 MHz, 100 MHz, and 1 MHz. As expected due to the tank circuit from both the external reader and the implant, no coupling between coils was observed at frequencies different from 10 MHz.

**Figure 13:**
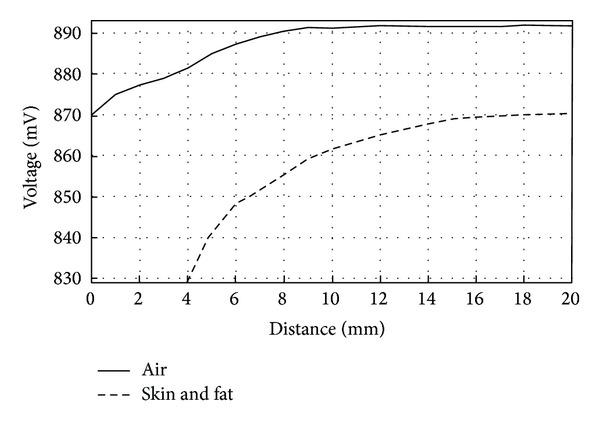
Voltage variations on reader coil versus distance. Transmission medium between coils: 1 mm of pork skin and 1 mm of fat.

In addition to the measurements mentioned above, measurements were made with two different transmission mediums. Mutual magnetic coupling between coils was measured by placing pork skin on top of the Maxwell-Wien bridge circuit. The skin consisted of 2 mm of skin and fat. The distance between coils was swept from 20 mm to 4 mm. The results depicted in Figure 13 show a larger variation in voltage when compared with air. Comparing these results with solely air as the transmission medium, this means that for a greater distance between coils, there still exists an influence in the reader coil due to the implant coil. In other words, magnetic coupling between coils was found to be better when the transmission medium included organic tissue.

According to the Wireless Power Consortium, typical coupling factor values for RFID applications are found to be in the range of 0.3 to 0.6 [26]. For our study, it was found that for a distance between coils of 5 mm, the coupling factor was of 0.2. For the latter reason the maximum distance between coils was established to be of 5 mm to ensure a proper magnetic coupling link, for a transmission medium of 1 mm of pork skin and 1 mm of fat.

**Figure 14:**
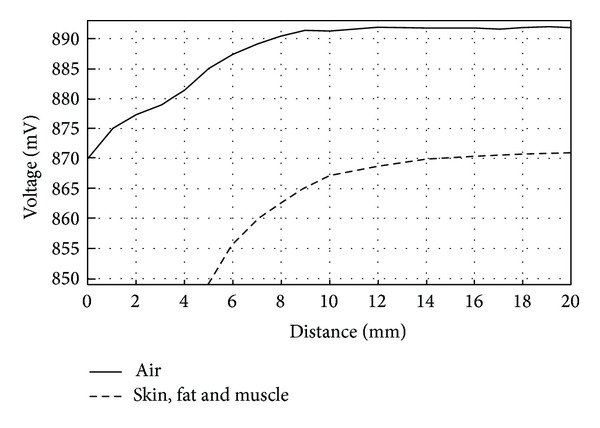
Voltage variations on reader coil versus distance. Transmission medium between coils: 1 mm of pork skin, 1 mm of fat, and 2 mm of muscle.

Magnetic coupling measurements between coils were repeated by placing 1 mm of pork skin, 1 mm of fat, and 2 mm of pork muscle over the Maxwell-Wien bridge circuit. The distance between coils was swept this time from 20 mm to 5 mm. As in the previous measurements, a larger variation in voltage was observed (Figure 14) when compared with only air as the transmission medium.

The effect of a larger variation in voltage observed in the measurements with organic tissue can be explained as follows: when the transmission medium was air, there was nothing between the coils. From the total radiated power of the reader coil, some of the power was lost in space and the rest was reflected by the implant coil. When organic tissue was placed above the reader coil, the transmission medium changed. In this case, again, from the total radiated power of the reader coil, some of the power was lost in space, but the reflected power to the reader coil was due to both the power reflected from the implant coil and to the power reflected from the tissue itself. The latter explains why when the coils are far apart from each other (9 mm for skin and fat tissue and 12 mm for skin, fat, and muscle tissue) the voltage variation is nearly the same as in the case of air as the transmission medium. It also explains the reason why when the coils are in close proximity, the voltage response is higher in both cases.

It was observed that when organic tissue was placed on top of the Maxwell-Wien bridge circuit, the voltage variation occurred earlier (at approximately 14 mm), as opposed to air as the transmission medium which occurred at a separation distance between coils of approximately 8 mm.

As with the transmission medium of 2 mm of skin and fat, in this case magnetic coupling between coils was found to be better when compared with air. Once the MEMS sensor is attached to the implant coil, experiments will be conducted to verify if the distance between coils can be increased without compromising the efficiency of the magnetic coupling link.

**Figure 15:**
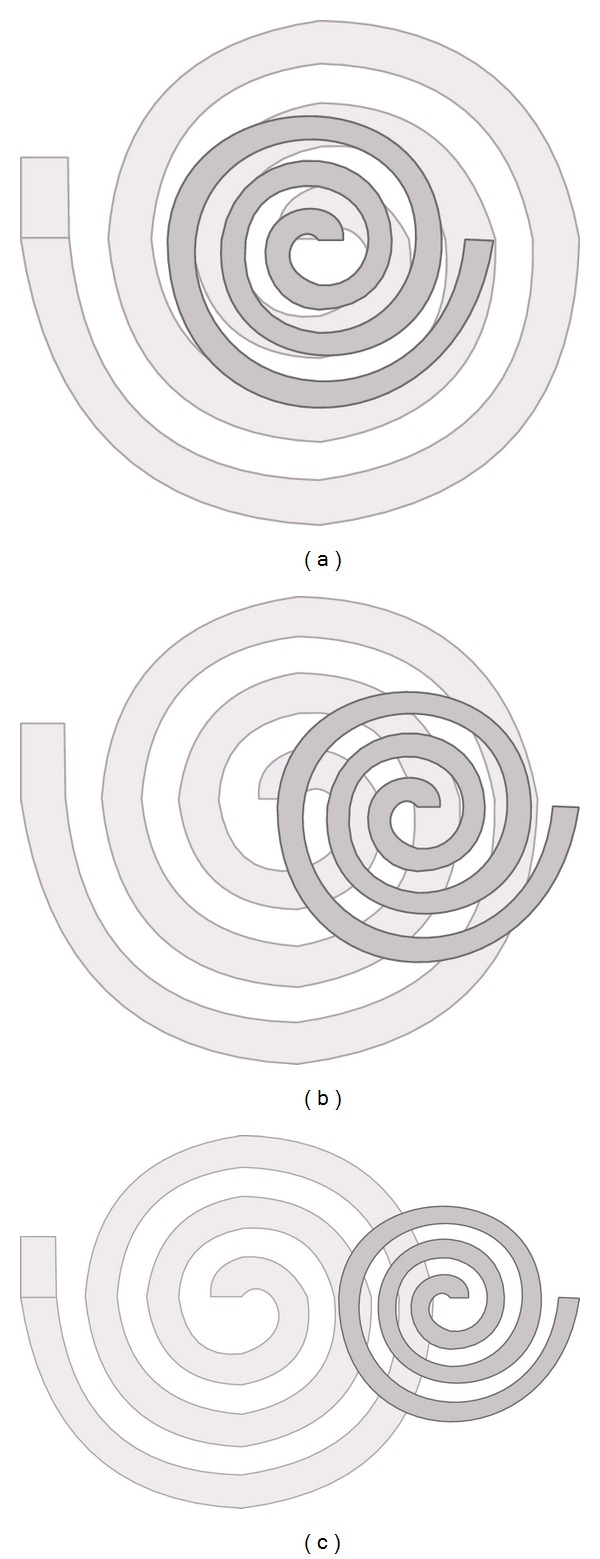
Misalignment between coils: (a) 0 mm, (b) 5 mm, and (c) 10 mm.

On the other hand, misalignment in coils can occur because their transverse axes cease to coincide [27, 28]. Although the inductors may remain parallel, a distance between the transverse axes of each coil can exist.  Figure 15 shows two inductors with 0 mm, 5 mm, and 10 mm of misalignment distance between them.

**Figure 16:**
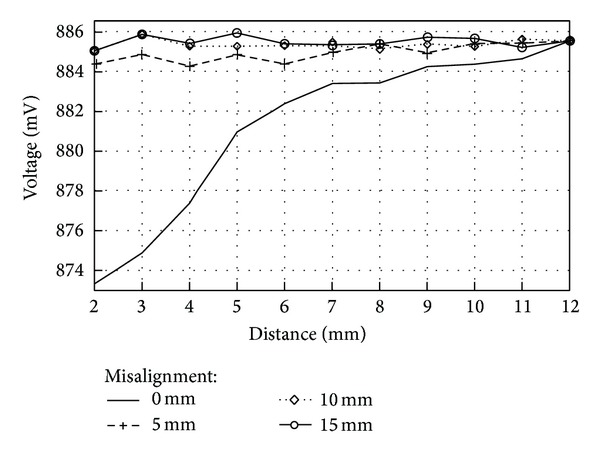
Voltage variations on reader coil versus distance. Transmission medium between coils: air.

Experiments to measure decoupling between coils due to misalignment were carried out by separating the transverse axes of the coils in 5 mm, 10 mm, and 15 mm units. For each of the misalignment distances mentioned earlier, peak voltage of the reader coil was measured at three different vertical distances between coils, 2 mm, 7 mm, and 12 mm units.  Figure 16 graphically summarizes the measurements made with air as the transmission medium.

It can be seen in Figure 16 that for a vertical distance between coils of 12 mm, misalignment played a minor role on magnetic coupling since all measurements converged to the same voltage value. As the distance between coils was reduced (7 mm and 2 mm for our case), however, it can be observed that misalignment distance becomes an important issue since voltage values for a vertical distance of 4 mm with a misalignment of 5 millimeters can be confused with voltage values for a vertical distance of 9 mm with zero misalignment.

**Figure 17:**
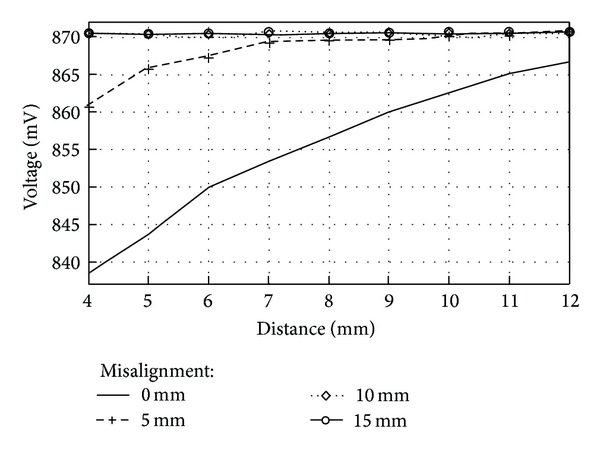
Misalignment between coils. Transmission medium between coils: 1 mm of pork skin and 1 mm of fat.

Since the measurements carried out with skin and fat tissue presented a greater voltage variation of 40 mV_p_, as opposed to the measurements with skin, fat, and muscle which presented a voltage variation of 20 mV_p_, misalignment measurements between coils were repeated by using skin and fat tissue. As with the former misalignment measurements, decoupling between coils was measured by separating the transverse axes of the coils at 5 mm, 10 mm, and 15 mm units, and for each of the misalignment distances, peak voltage of the reader coil was measured at three different vertical distances between coils, 4 mm, 7 mm, and 12 mm.  Figure 17 shows the measurements made with a transmission medium of 1 mm of pork skin and 1 mm of fat.

It can be seen from Figure 17 that measurement results were very similar to those with only air as the transmission medium. For a misalignment distance of 5 mm, coupling between coils still occurred, but for a misalignment distance of 10 and 15 mm, there was no coupling between coils. This is why it was determined that the maximum distance of misalignment between coils was 5 mm, because at 5 mm there still existed coupling between coils. At 10 mm of misalignment or further, no coupling between the coils occurred so there is no way a magnetic coupling link could exist.

In order to obtain the best power transmission and the lowest measurement error it is, thus, of critical importance to place the coils as close to each other as possible and to guarantee that the coil of the implant is within the area of the reader coil. One way to achieve this is to make the area of the reader coil larger than the area of the implant coil.

### 3.3. New Prototypes

**Figure 18:**
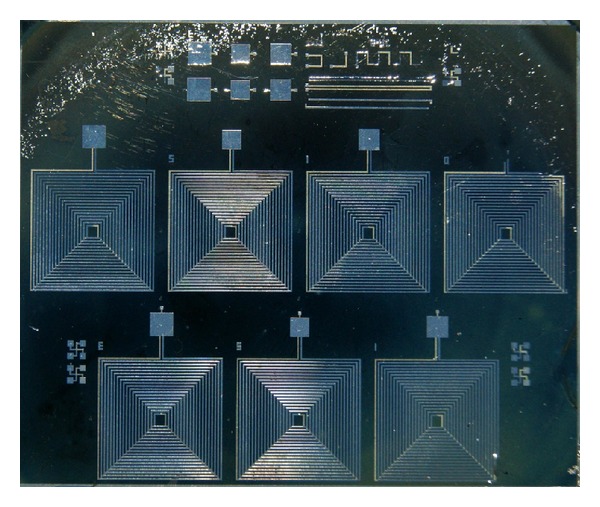
RLC circuits encapsulated in polyimide.

**Figure 19:**
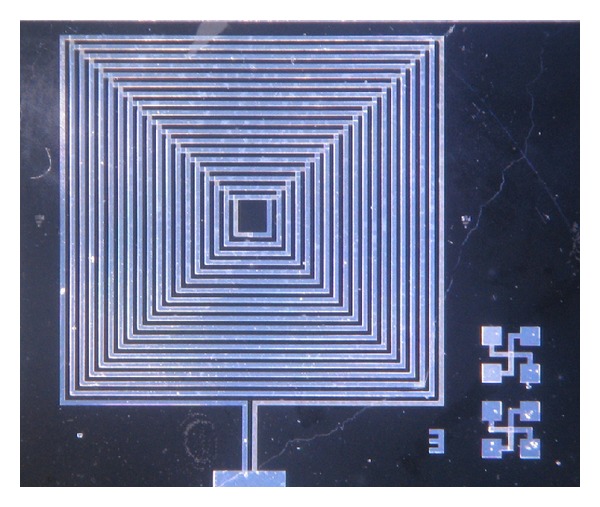
Coil layout.

New prototypes of the implant RLC circuit were fabricated at the National Institute of Astrophysics, Optics and Electronics with a two-metal layer fabrication process. The fabricated RLC circuits are shown in Figure 18. The metal used in the fabrication process was aluminum. The capacitor consisted of two parallel plates of 1 *μ*m in thickness each and with 1.5 *μ*m of polyimide as the dielectric material between them. The second capacitor from right to left of the upper row of coils in Figure 18 was designed to have a capacitance of 32 pF. The last two capacitors from the left of the same upper row were designed to have a −10% and +10% of area from the original capacitor of 32 pF, respectively. Pads to the lower row of RLC circuits in the layout were added to measure capacitance and electrical resistance (Figure 19). Additional test structures were added to measure contact and resistance between metal layers.

Taking advantage of the 2-metal layer fabrication process, the coil was designed with two layers of metal in order to obtain a greater value of inductance. Each coil is 8 mm per side, with 18 turns each coil. The aluminum tracks are 100 microns wide and the distance between tracks is 100 *μ*m also. The value obtained from simulations for the inductance value was 7.84 *μ*H, with a resistance of 149.9 Ω and a *Q* value of 3.28.

With the calculated values for both the capacitor and the coil it is expected that the RLC circuit will have a resonance frequency of 10 MHz. The new prototypes of the implant will serve to lead biocompatibility and wear tests of polyimide. Rabbits will be used to conduct the trials mentioned above for as long as three months, in the facilities of the Association to Avoid Blindness in Mexico. Power transmission efficiency tests will also be performed with different transmission mediums [29] and the experiments carried out in this study will be repeated with the new prototypes to compare the efficiency of the inductive link.

## 4. Conclusions

A wireless energy and data transfer system for intraocular pressure measurement was presented. The implant was encapsulated in a polyimide coating for biocompatibility with the human body. The RLC passive circuit proposed in the implant minimizes power consumption, lowering the risk of tissue damage due to overheating and because of the intrinsic nature of the circuit, it filters out undesired coupling with other radiating sources at different frequencies thereby, reducing possible errors associated with the readings. The frequency for power and data transmission was chosen to be 10 MHz, which complies with the Mexican Federal Communications Commission regulation for free public radioelectrical radiation.

The experiments presented in this paper were conducted emulating a more realistic environment by introducing organic tissue between the coils. 1 mm of pork skin and 1 mm of fat were used in one case and 1 mm of pork skin, 1 mm of fat, and 2 mm of muscle were used in the other.

The measurement system proposed in this paper presented a tolerance of 5 mm as the maximum misalignment distance, with a transmission medium of 1 mm of skin and 1 mm of fat. For the same transmission medium and for a misalignment distance between coils of 0 mm, the maximum distance between coils for a proper power transfer was found to be 5 mm.

The system presented in this paper is suitable for energy transfer in intraocular pressure measurement applications. Special care must be taken when placing the two coils in close proximity to avoid misalignment between them. In order to have the maximum power transfer between coils, a design methodology that optimizes inductive power transmission must be chosen [30].

### 4.1. Future Work

Future work will focus on interconnecting the coil and the MEMS sensor of the implant and conduct power transmission efficiency tests with different transmission mediums. It is expected that the RLC circuit of the implant fabricated with MEMS technology will solve current technological issues in intraocular pressure measurement such as taking erroneous measurements by placing the sensor in direct contact with the aqueous humor while keeping the coil over the sclera.

## Figures and Tables

**Table 1:** Values of the components from the Maxwell-Wien bridge circuit.

	Component name on circuit	Value	Units
Resistor	*R*_2_, *R*_4_	50	Ω
Capacitor	*C*_1_, *C*_*x*_	2.7	nF
Inductor	*L*_1_, *L*_*x*_	90.93	nH
	Number of turns	3	—
	Internal radius	1	mm
	Conductor width	1.75	mm
	Conductor spacing	1.75	mm
	Total diameter	23	mm
